# Biochemical changes in *Robinia pseudoacacia* leaflets in dependence of leaf mining, plant age and location

**DOI:** 10.1186/s12870-026-08364-6

**Published:** 2026-02-14

**Authors:** Svitlana Sytnyk, Rabea Schweiger, Kyrylo Holoborodko, Caroline Müller

**Affiliations:** 1 Bielefeld University, Universitätsstraße 25, Bielefeld, 33615 Germany; 2 Dnipro State Agrarian and Economic University, S. Yefremov Str. 25, Dnipro, 49009 Ukraine; 3 Joint Institute for Individualisation in a Changing Environment (JICE), University of Münster and Bielefeld University, Bielefeld, Germany; 4 Oles Honchar Dnipro National University, Nauky Ave. 72, Dnipro, 49045 Ukraine

**Keywords:** Allocation, Carbon, Gracillariidae, Herbivory, Leaf miners, Metabolomics, Nitrogen

## Abstract

Plant responses to leaf-mining insects are still poorly studied, especially in woody species. *Robinia pseudoacacia* is widely cultivated in Ukraine, where it is not native. There, the trees are facing increasing damage from the likewise introduced leaf miner species *Parectopa robiniella* and *Macrosaccus robiniella*. We examined the effects of infestation by these species on foliar carbon (C) and nitrogen (N) content and on metabolic fingerprints of trees of different age and at different locations, expecting context dependency in induction responses. Uninfested or miner-infested leaflets were collected from infested trees of three age classes at one location and from trees of one age class at four distinct locations. C and N content were mostly determined by location, with minor effects of tree age and herbivory, for N in interaction. Among the 3,121 metabolic features detected with UHPLC-QTOF-MS/MS, 1,087 were quantitatively modulated by herbivory in at least one age–location combination. Metabolic responses to the two leaf miner species partially overlapped. The magnitude and direction of metabolic shifts varied with both tree age and location. The differences in leaf biochemistry and in responses to herbivory between tree age classes and locations may reflect physiological constraints, past environmental conditions, age-dependent defense strategies, resource availabilities and allocation as well as local edaphic and microclimatic conditions, but potentially also different damage levels. That the leaflets of *R. pseudoacacia* exhibited context-dependent biochemical responses to the leaf miners highlights the importance of accounting for plant ontogenetic stage and local environmental heterogeneity when evaluating plant–leaf miner interactions.

**Supplementary Information:**

The online version contains supplementary material available at 10.1186/s12870-026-08364-6.

## Background

Plant biochemistry is shaped by a wide array of ecological factors, including both biotic and abiotic ones [1]. Induced chemical defense responses of plants against (ectophagous) leaf-chewing insects are well characterized [2, 3] and may be influenced by factors such as plant ontogeny [4] and abiotic conditions [5, 6]. In contrast, plant responses to damage caused by leaf miners, i.e., endophagous herbivores that feed within epidermal and/or mesophyll tissues, have received comparatively less attention [7, 8]. Induced defense responses are particularly interesting to study in trees due to their long lifespans [9–11]. Introduced tree species growing outside their native range may develop different induced defense responses shaped by novel ecological pressures in their new environments [12, 13]. A better understanding of these mechanisms is crucial for predicting how non-native tree species may deploy defense strategies and adapt to novel ecosystems.

Leaf miners can manipulate the nutritional value of their host plant, modulating source-sink relationships [8]. In leaves of the mangrove *Avicennia marina* (Acanthaceae), mining in the epidermis by *Phyllocnistis citrella* (Lepidoptera: Gracillariidae) resulted in reduced contents of carbon (C) in the mined parts and enhanced contents of nitrogen (N) in non-mined parts of mined leaves compared to non-mined leaves [14]. Moreover, mining by insects causes changes in the composition of various specialized metabolites [15]. For example, in leaves of tomato (*Solanum lycopersicum*, Solanaceae), attack by *Tuta absoluta* (Lepidoptera: Gelechiidae) led to both local and systemic changes in various metabolites, especially an increase in phenolamides, with the local responses being much more pronounced [16]. Leaves of chickpea (*Cicer arietinum*, Fabaceae) infested by leaf miners showed enhanced concentrations of total phenols and flavonoids [17]. In apple trees (*Malus domestica*, Rosaceae), leaf-mining by *Phyllonorycter blancardella* (Lepidoptera: Gracillariidae) prevented the mobilization of phenolic compounds by the trees [8]. Leaf miner-induced metabolic changes may partly result from an accumulation of cytokinins in the mined tissues, which may prevent early leaf senescence [18]. In how far plant responses are specific for different miner species is, to our knowledge, unknown.

Moreover, induced responses to wounding may be highly context-dependent, for example with regard to plant age and in dependence of the environment [19, 20]. Trees of different age may encounter different combinations of environmental challenges throughout their ontogeny, for example, in terms of water and light availability, and therefore differ in their responses to stresses such as herbivory [11]. With increasing tree age and thus size, C pools usually increase in storage organs [11], but this may not necessarily be reflected in the leaves. When sampling leaves from two tree species and of different age along an elevational gradient, both saplings and adult trees showed consistent C and N allocation responses to elevation [21]. In contrast, a meta-analysis revealed that both C and N accumulate in aboveground plant parts with increasing forest stand age (i.e., within the first 30 years) but eventually reach an equilibrium state [22]. Likewise, the concentrations of defense metabolites may change with tree age. For example, concentrations of low-molecular-weight phenolics and monoterpenoids in leaves were found to decline with tree age, while condensed tannins increased [23–25]. Leaf traits may also become more diverse with plant age [25]. These patterns may also vary due to different environmental conditions, such as nutrient availability [11, 26]. Moreover, when plants expand their ranges and become invasive, allocation in defenses may shift, for example, due to changes in selection pressures by distinct herbivore communities [27, 28].

*Robinia pseudoacacia* (black locust, Fabaceae) is a fast-growing woody plant species native to North America, that has been introduced to most other continents and is considered invasive in many countries [29]. It is regarded a valuable but problematic plant species due to its positive and negative environmental effects. Planted on purpose in European plantations, *R. pseudoacacia* plays an important economic role and performs essential ecosystem services, such as C sequestration and soil erosion protection [30]. However, this species also outcompetes native plants and changes soil chemistry [29]. As a Fabaceae, it is capable of atmospheric nitrogen fixation through symbiosis with rhizobia, potentially improving its N availability [31]. Numerous insect species are herbivorous on *R. pseudoacacia* and some of these species were introduced to Europe [32]. In Ukraine, the two leaf-mining, invasive species *Parectopa robiniella* and *Macrosaccus robiniella* (previously known as *Phyllonorycter robiniella*) (both Lepidoptera: Gracillariidae) infest *R. pseudoacacia* [33]. The former species is known to impact the photosynthetic apparatus [34]. The chemistry of *R. pseudoacacia* is characterized by various phenolics, terpenoids and different lectins [35–37]. However, little is known about potential changes of plant metabolites in this plant species caused by leaf miners and how specific these responses are regarding the mining species.

To gain insights in the specificity of metabolic changes in *R. pseudoacacia* in dependence of different factors, i.e., herbivory by the two miner species, plant age and plant community location, we sampled leaf material, determined its C and N content and applied metabolic fingerprinting. Uninfested leaflet sections of uninfested or miner-infested compound leaves of miner-infested trees were collected from trees of different age at one location as well as from trees of one age class from four locations. We expected that the C and N content as well as the composition of metabolites differ between trees of different age classes and between locations, as the latter ones show distinct edaphic and other environmental conditions. Moreover, we assumed that these biochemical traits are affected by herbivory in an herbivore species-specific manner, with the responses depending on tree age. Differences in leaf chemistry in response to leaf mining were also expected to some extent between the locations.

## Materials and methods

### Study locations

The samples were collected from *R. pseudoacacia* growing in Dnipro city and its vicinity (Supplementary Fig. S1) in the Ukrainian North Steppe subzone, which has a temperate continental climate. Four study locations were selected (BotG, Botanical Garden; May, Mayorka; MonIs, Monastyrsky Island; LomFP, Lomyvsky Forest-Park), which are characterized by different environmental conditions and in which *R. pseudoacacia* trees serve distinct functions, either as ornamental plantations in green recreational zones or to protect against soil erosion (Supplementary Table S1).

The formal taxonomic identification of *R. pseudoacacia* was carried out by Dr. Iryna Ivanko. Voucher specimens of this species have been deposited in the Herbarium of Oles Honchar Dnipro National University (DSU), Ukraine, voucher numbers 123,017–123,119.

### Sampling

We sampled biomass from urban trees for which the exact planting origin cannot be determined; no seeds or reproductive material were used. As these trees are components of municipal urban green infrastructure, no special permits were required for sample collection. Leaflets of *R. pseudoacacia* plants were collected during the vegetation season in September 2024. In the study region, the studied leaf miner species are usually in their second generation (personal observation). For the sampling, trees younger than 10 years with similar morphological and biometric characteristics were selected per location. In the Botanical Garden, samples were taken in addition from trees of 10–25 years and trees older than 25 years. Trees were selected based on similar characteristics within age class. Tree cores were taken at a height of 1.3 m of the stems to estimate the age of each tree. At each site, leaf samples of 10 trees were taken, except for Monastyrsky Island, where only five trees of the respective category were available and these were sampled twice. In total, there were 16 groups (Supplementary Table S1).

All trees showed symptoms of herbivore damage by either one or both miner species, *P. robiniella* (P) and *M. robiniella* (M). While both herbivore species consume mesophyll and produce blotch mines, the spatial feeding patterns slightly differ. Larvae of *P. robiniella* feed digitate mines closer to the adaxial leaf side, while larvae of *M. robiniella* form elongate-oval mines at the abaxial leaf side [38, 39] (Fig. S2). Trees infested with P and trees infested with P and M were selected for sampling. Within these trees, terminal leaflets of one of three categories were collected: without damage symptoms (“uninfested”, U; Fig. S2b), infested with P (Fig. S2c) or infested with M (Fig. S2d). For the category U, the remaining parts of the compound leaves were uninfested, while for the categories P and M other leaflets of the compound leaves were uninfested or infested by the same herbivore species. Leaf samples were taken under cloudless conditions from the lower third of the crown of southern exposure at approximately the same sampling height (1.7–1.9 m) across trees. First, 20 terminal leaflets per tree from entire compound leaves of the same infestation category were harvested using scissors, washed with tap water and dried on paper sheets at ambient temperature within about 5–7 min. Then, only the upper section of the terminal leaflet of each compound leaf was cut with a scalpel, avoiding the lower section which carried the mines in case of infested leaflets. Leaflet sections from 20 leaves were pooled in one paper bag, representing one sample. All stages of the sampling were carried out with gloves and the instruments used (scissors, scalpel) were cleaned with ethanol between samples. The leaflets were air-dried at ambient conditions in the dark for 6–7 weeks and further dried in a drying oven at 40 °C for 4 d. A similar approach had been previously used by other researchers to analyze various phytochemicals from *R. pseudoacacia* leaves that had been air-dried at room temperature [35]. Sampling in liquid nitrogen or direct quenching in solvents was logistically not possible in the present study.

### Chemical analyses

For chemical analyses, the samples were milled, processed and analyzed in random order. The carbon (C) and nitrogen (N) contents in the samples (4 mg dw) were assessed with a C/N analyzer (Vario MICRO Cube, Elementar Analysensysteme, Hanau, Germany). Metabolic fingerprinting of (semi-)polar metabolites was performed as described in [40] with some modifications. Leaf powder (10 mg dw) was extracted in an ice-cold ultrasonic bath for 15 min, using 450 µL ice-cold 90% methanol (LC-MS grade, Thermo Fisher Scientific, Loughborough, UK) with 10 mg L^− 1^ hydrocortisone (Sigma-Aldrich, Steinheim, Germany) as internal standard. Twelve blanks without plant material were prepared in addition. Supernatants were filtered (0.2 μm, Phenomenex, Torrance, CA, USA) and subjected to ultra-high performance liquid chromatography (UHPLC: Dionex UltiMate 3000, Thermo Fisher Scientific, San José, CA, USA) coupled to quadrupole time-of-flight mass spectrometry (QTOF-MS/MS: compact, Bruker Daltonics, Bremen, Germany). Chromatographic separation of samples (4 µL) was performed at 45 °C on a Kinetex XB-C18 column (150 × 2.1 mm, 1.7 μm, with guard column, Phenomenex), using the following gradient involving eluent A [H_2_O with 0.1% formic acid (FA; Sigma-Aldrich)] and eluent B [acetonitrile: LC-MS grade (Thermo Fisher Scientific); with 0.1% FA] at a flow rate of 0.5 mL min^− 1^: 2 to 30% B within 20 min, to 75% B within 9 min, followed by cleaning and equilibration of the column. Via a T-piece, part of the sample stream was subjected to negative electrospray ionization and QTOF-MS/MS, with the following settings in MS mode: 5 Hz spectra rate, centroid mode, mass-to-charge (*m*/*z*) range 50–1,300, end plate offset 500 V, capillary voltage 3000 V, N_2_ as nebulizer (3 bar) and dry gas (12 L min^− 1^, 275 °C), low mass *m*/*z* 90, quadrupole ion energy 4 eV, collision energy 7 eV. The AutoMSMS mode was used for fragmentation (MS/MS), with N_2_ as collision gas and ramping isolation widths and collision energies along with the precursor *m*/*z*. Prior to each sample, sodium formate solution was pumped into the QTOF for *m*/*z* recalibration. Some samples had to be excluded due to technical issues, reducing the samples sizes of the LC-MS dataset to *n* = 7–10.

The T-ReX 3D algorithm (MetaboScape 2021b, Bruker Daltonics) was used for *m*/*z* axis recalibration and for picking of metabolic features, each being described by a retention time (RT) and *m*/*z* value, in MS mode. Features with a minimum peak height of 1,000 and at least 15 data points were included. All features likely belonging to the same metabolite were collected in so-called buckets, using a correlation coefficient threshold of 0.8 and allowing [M–H]^–^, [M + Cl]^–^, [M+HCOOH–H]^–^, [2 M–H]^–^ and [M–H–H_2_O]^–^ ions along with their corresponding isotopes and charge states. For quantification, from each bucket the feature with the highest intensity was used, omitting features within or close to the injection peak (i.e., those with RT < 1.25 min). Peak heights were divided by those of hydrocortisone ([M+HCOOH–H]^–^), the averaged blanks were subtracted and peaks were divided by the sample mass.

### Data analyses

If not stated otherwise, data analyses were done in R (4.4.2 [41]). A significance threshold of α = 0.05 was applied (and α < 0.1 for marginal significance). According to the research questions stated above, the data were split into data-subsets. To investigate the effects of tree age class and herbivory, only data from the Botanical Garden were included (data-subset I), comprising the three age classes (< 10 years, 10–25 years, > 25 years) as well as the three herbivory groups (U, P, M). For evaluation of the impacts of location and herbivory, only data from trees with an age of < 10 years were included, with data-subset IIa containing two locations (May, MonIs) with two herbivory groups (U, P) and data-subset IIb containing two locations (BotG, LomFP) with three herbivory groups (U, P, M). The C and N contents were visualized as box-whisker plots (“boxplot”, R package *graphics*) and analyzed with (generalized) linear models (R package *glmmTMB*), with the fixed factors age class and herbivory (data-subset I) or location and herbivory (data-subsets IIa, IIb) as well as their interaction term. Tree identity was not included as random intercept, as it led to model singularities. The R packages *DHARMa* and *performance* were used for the evaluation of goodness of model fit and model diagnostics. For the N content in data-subset IIb, inverse group variances were used in a weighted least squares model (“lm”, R package *stats*) to account for heteroscedasticity. Type II Wald *Χ²*-tests were used to derive *p*-values (“Anova”, R package *car*). If there were significant factors or interaction terms, manual contrasts with Bonferroni-Holm adjustment were computed (R package *emmeans*). For evaluation of differences in C and N contents between trees of varying age (data-subset I), we compared uninfested (U) leaflets of trees with an age of 10–25 years with leaflets from younger trees (< 10 years) and, in a second contrast, with those of older trees (> 25 years). To compare locations, we contrasted uninfested leaflets between locations (data-subsets IIa and IIb). To assess responses to herbivory, leaflets of each herbivory group (P, M) were compared to uninfested leaflets for each age class and location (all data-subsets). To compare metabolic fingerprints between samples, non-metric multidimensional scaling (NMDS) analyses with Wisconsin double standardization of square root-transformed data and Kulczynski distances were conducted (R package *vegan*), across all samples as well as separately for each data-subset. The effects of herbivory within tree age classes and locations were further traced, separately comparing the groups P and M with the control (U). To quantify metabolic responses to herbivory and compare them across tree age classes and locations, for features that occurred in both groups, mean fold changes (FC) were calculated as mean feature intensities in the samples from herbivore-infested leaves divided by the mean feature intensities in the corresponding control samples (P/U, M/U). All features showing a log_2_ fold change of < -1 or > 1 in at least one pairwise comparison (i.e., features quantitatively “modulated” by herbivory, see below) were used to construct a cluster heatmap (average linkage, hierarchical, Pearson distances), clustering both over the metabolic features as well as the age class * location combination across all tree ages and locations (Cluster 3.0 [42]; Java TreeView 1.1.6r4 [43]). Metabolic features were considered as being quantitatively modulated by herbivory, if they were found in both groups with a log_2_ FC of < -1 (original scale: < 0.5; lower under herbivory) or > 1 (original scale: > 2; higher under herbivory) and as qualitatively modulated if they occurred in at least half of the samples of one group and were absent in the other group. The overlaps of these modulated features between tree age classes, locations and the herbivory groups were depicted as Venn diagrams (R package *gplots*).

## Results

### C and N contents

**Fig. 1:**
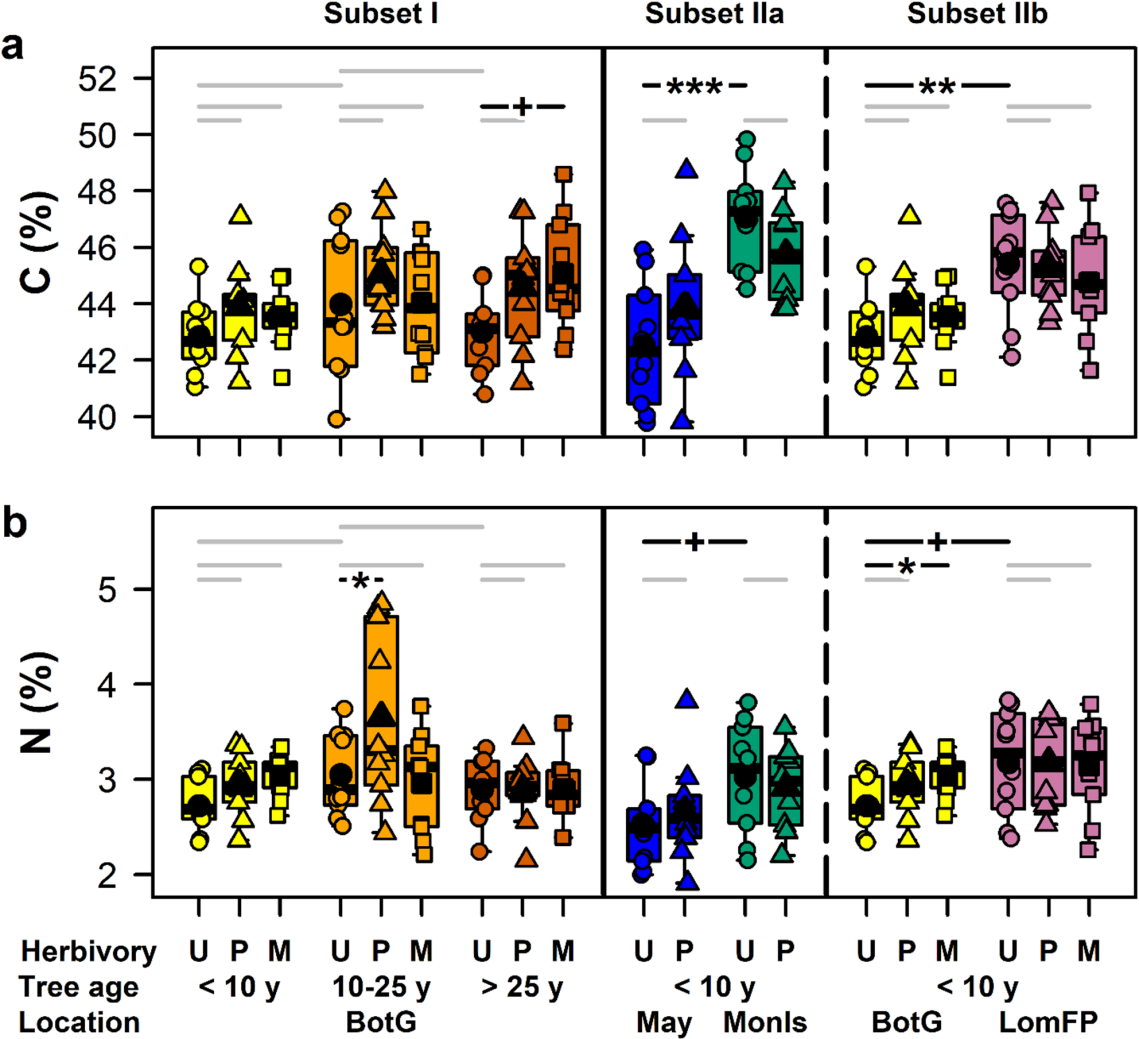
Effects of herbivory, tree age and location on carbon and nitrogen contents of *Robinia pseudoacacia* leaflets. **a** Carbon (C) contents. **b** Nitrogen (N) contents. Data are presented in dependence of tree age and herbivory (data-subset I with data from Botanical Garden only; left) or location and herbivory (data-subset II with tree ages < 10 years only; right). The data are shown as box-whisker plots with interquartile ranges (IQR, lower to upper hinge) as boxes, medians as horizontal thick lines, means as large black symbols, whiskers extending to the lowest and highest data points within 1.5 times the IQR and raw data points as small symbols. For different locations (BotG, Botanical Garden; May, Mayorka; MonIs, Monastyrsky Island; LomFP, Lomyvsky Forest-Park) and tree age classes, data from uninfested leaflets (U) as well as from leaflets infested with *Parectopa robiniella* (P) or *Macrosaccus robiniella* (M) are given. For statistical tests, see Table 1; if factors or interactions were significant, manual contrasts were computed and are shown as horizontal lines above the graphs [gray, not significant; black, (marginally) significant with + *p* < 0.1, * *p* < 0.05, ** *p* < 0.01, *** *p* < 0.001]; *n* = 10

The C content of *R. pseudoacacia* leaflets ranged from 39.8% to 49.8% (Fig. 1a). In the Botanical Garden (data-subset I), it was slightly (marginally significant, *p* < 0.1) affected by tree age and significantly by herbivory (Table 1). For trees with an age of > 25 years, leaflets infested with *M. robiniella* showed slightly (marginally significant) higher C contents than uninfested leaflets. Furthermore, the C content was significantly affected by the interaction between location and herbivory (data-subset IIa) and by location (data-subset IIb) (Table 1). Trees from Monastyrsky Island and Lomyvsky Forest-Park showed higher C contents of uninfested leaflets than trees from Mayorka and the Botanical Garden, respectively. The foliar N content ranged from 1.9% to 4.9% (Fig. 1b). It was significantly influenced by the interaction between tree age and herbivory (Botanical Garden, data-subset I; Table 1). In trees with an age of 10–25 years, the N content was significantly higher in *P. robiniella*-infested than in uninfested leaflets. It was also significantly impacted by location and, for data-subset IIb, slightly (marginal significance) by herbivory (Table 1). The N content was higher (marginal significance) in uninfested leaflets of trees from Monastyrsky Island and Lomyvsky Forest-Park compared to those from Mayorka and the Botanical Garden, respectively. In data-subset IIb, leaflets infested with *M. robiniella* showed a significantly higher N content than uninfested leaflets within the Botanical Garden.

**Table 1:** Results of statistical models for carbon (C) and nitrogen (N) contents of *Robinia pseudoacacia* leaflets

Data subset	Response variable	Error distribution (link function)	Fixed factors	Test statistic		df	*P* ^1^
I	C content	Gaussian (identity)	tree age (A)	*Χ²*	5.46	2	*0.065*
			herbivory (H)	*Χ²*	9.06	2	**0.011**
			A x H	*Χ²*	4.14	4	0.388
	N content	Gamma (log)	tree age (A)	*Χ²*	11.32	2	**0.003**
			herbivory (H)	*Χ²*	6.04	2	**0.049**
			A x H	*Χ²*	11.44	4	**0.022**
IIa	C content	Gaussian (identity)	location (L)	*Χ²*	27.68	1	**< 0.001**
			herbivory (H)	*Χ²*	< 0.01	1	0.991
			L x H	*Χ²*	4.83	1	**0.028**
	N content	Gaussian (identity)	location (L)	*Χ²*	6.87	1	**0.009**
			herbivory (H)	*Χ²*	0.02	1	0.899
			L x H	*Χ²*	0.82	1	0.366
IIb	C content	Gaussian (identity)	location (L)	*Χ²*	20.84	1	**< 0.001**
			herbivory (H)	*Χ²*	1.16	2	0.561
			L x H	*Χ²*	2.78	2	0.249
	N content	Gaussian (identity), weighted	location (L)	*F*	5.56	1	**0.022**
			herbivory (H)	*F*	3.08	2	*0.054*
			L x H	*F*	0.89	2	0.415

^1^Significant *p*-values (< 0.5) are indicated in bold, marginally significant ones (< 0.1) in italics

### Metabolic fingerprints

**Fig. 2:**
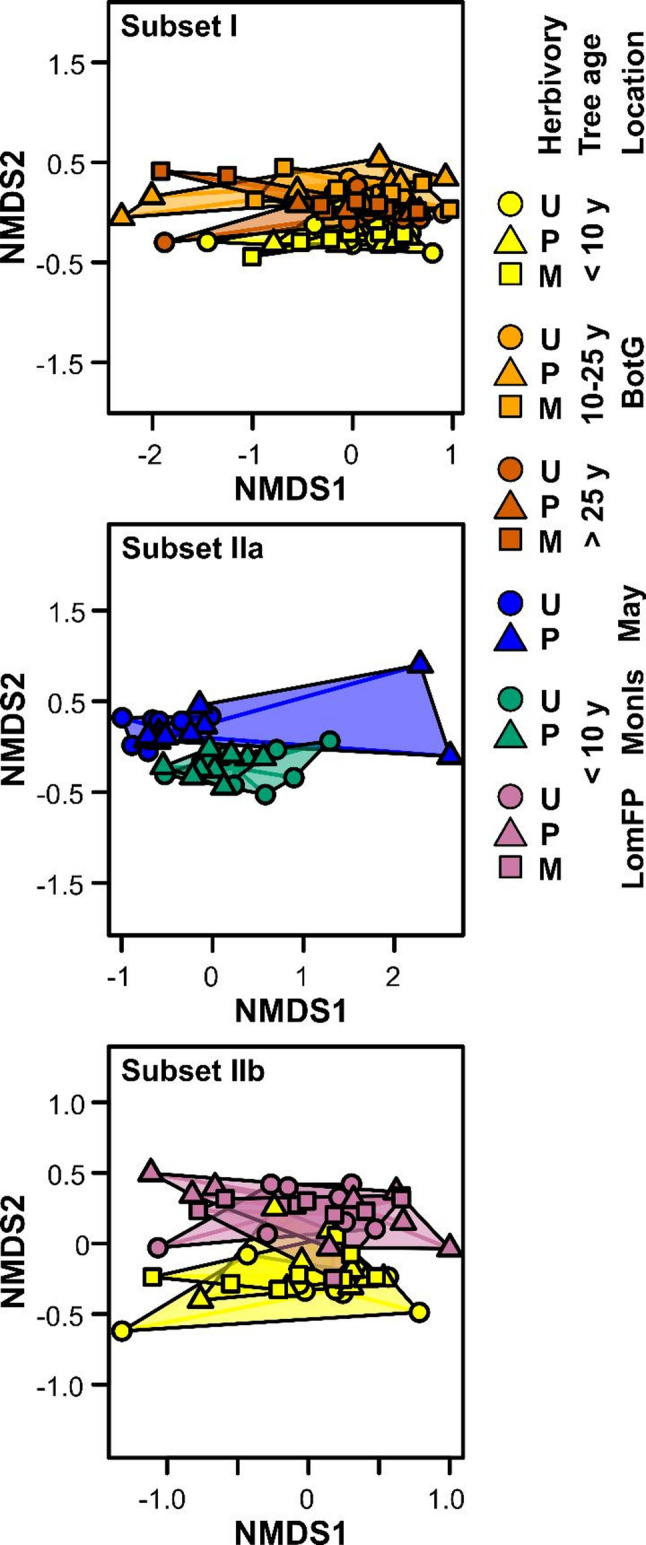
Metabolic composition of *Robinia pseudoacacia* leaflets in depen-dence of leaf herbivory, age class and location. Data are presented as non-metric multidimensional scaling (NMDS) plots. Leaflets were uninfested (U) or infested by herbivores [*Parectopa robiniella* (P), *Macrosaccus robiniella* (M)]. Data are shown for different tree age classes and locations (BotG, Botanical Garden; May, Mayorka; MonIs, Monastyrsky Island; LomFP, Lomyvsky Forest-Park), with data-subset I containing data from the Botanical Garden with different age classes (2,307 metabolic features, stress value 0.093; top) and data-subsets IIa (2,079 features, stress value 0.053, middle) and IIb (2,016 features, stress value 0.124, bottom) encompassing young (< 10 years) trees of different locations. The groups are surrounded by convex hulls and scores are connected to the group medians; *n* = 7–10

**Fig. 3:**
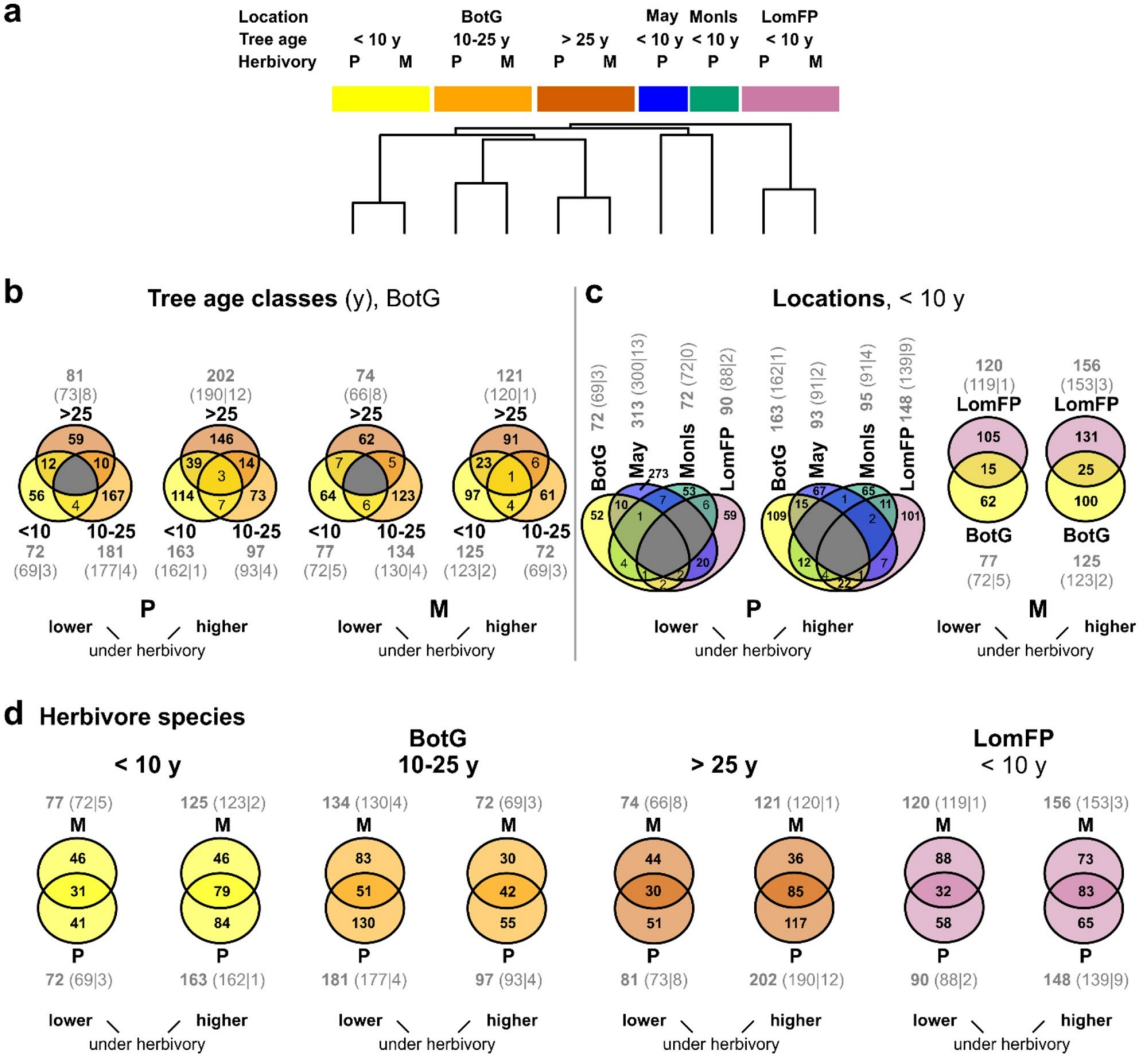
Metabolic responses of *Robinia pseudoacacia* leaflets to herbivory. For different tree age classes and locations (BotG, Botanical Garden; May, Mayorka; MonIs, Monastyrsky Island; LomFP, Lomyvsky Forest-Park), responses to infestation by *Parectopa robiniella* (P) or *Macrosaccus robiniella* (M) are shown. **a** Cluster analysis using mean fold changes (log_2_-scaled, based on *n* = 7–10) of metabolic features in herbivore-infested leaflets compared to uninfested leaflets. The analysis is based on those 1,087 metabolic features, which occurred in both groups and were quantitatively modulated (log_2_ fold change < -1 or > 1) in at least one pairwise comparison. For the corresponding heatmap, see Fig. S4. In **b**-**d**, the number and overlap of features that were modulated by the two herbivores are shown as Venn diagrams. Overlap of responses to a given herbivore species between tree age classes (BotG; **b**) and locations (tree ages < 10 years; **c**). **d** Overlap of responses to the two herbivore species, within age classes and locations. Features were considered as being modulated by herbivory, if they were found in both groups with a log_2_ fold change of < -1 (lower under herbivory) or > 1 (higher under herbivory) or if they occurred in at least half of the samples of one group and were absent in the other group (i.e., quantitative respective qualitative modulation). Within each comparison, the features that had lower concentrations or were not found under herbivory are shown on the left, while those that had higher concentrations or were only found under herbivory are shown on the right. Empty intersections (no features) are shown in gray, intersections with at least 10 features are given in bold. Gray numbers indicate the number of features, which were modulated by herbivory (total number in bold, quantitative|qualitative in parentheses) for each pairwise comparison; data based on *n* = 7–10

In total, 3,121 metabolic features were found in the *R. pseudoacacia* leaflets. Each metabolic feature is characterized by a RT and *m*/*z* value and most of the features probably represent distinct metabolites. The metabolic composition of the leaflets partly overlapped between the groups (Figs. 2, S3). While the metabolic fingerprints showed some variation with tree age and differed between locations, effects of herbivory on the overall metabolic composition were less pronounced (Fig. 2). However, further tracing metabolic responses to herbivory within age classes and locations revealed various metabolic features that were affected by herbivory. Indeed, 1,087 features were quantitatively modulated by herbivory by at least one herbivore species in at least one tree age * location group, partly having lower and partly having higher concentrations under herbivory (for all log_2_-scaled fold changes, see Table S3). Clustering of fold changes showed that the metabolic responses to herbivory mainly differed between locations and, for the Botanical Garden, between tree age classes, while responses to the two herbivore species were more similar (Figs. 3, S4). Compared to the quantitatively modulated features, only few features were qualitatively modulated by herbivory, i.e., only occurred in uninfested or in herbivore-infested leaflets of a given pairwise comparison (Fig. 3b-d). Leaflets of trees at Mayorka were most responsive to herbivory, with 406 metabolic features (313 lower, 93 higher) being modulated by *P. robiniella* infestation, while leaflets from Monastyrsky Island were least responsive (167 features modulated by *P. robiniella*: 72 lower, 95 higher). The overlapping response analysis revealed quite distinct metabolic responses of trees of the three age classes (Fig. 3b) and of the four locations (Fig. 3c) to herbivory. While many metabolic responses were specific for one herbivore species (P or M), there were also quite large overlaps in responses to the two herbivore species (Fig. 3d).

## Discussion

In line with our expectations, both the C and N content as well as the composition of plant metabolites were affected by the leaf miners. However, there was only a slight impact of tree age on C and N contents, but there were pronounced differences in C and N contents between the locations. The two miner species caused partly distinct but also shared metabolic shifts. More pronounced differences in metabolite composition were found among locations but also based on tree age.

The ranges of the C and N contents observed in our study for *R. pseudoacacia* leaflets are in line with previously reported values for leaves from trees of comparable age but at other locations [44–46]. With regard to age, slight impacts on C content but stronger effects on N content were found for plants from the Botanical Garden. In *R. pseudoacacia* trees in China, significant increases in C and N were reported along with increasing age from 10 to 36 years, followed by slight decreases up to an age of 45 years [45]. Structural substances with higher amounts of C may accumulate in middle-aged trees under certain conditions [45]. In our study, leaflets of *R. pseudoacacia* trees from Monastyrsky Island exhibited higher foliar C and N contents compared to those from the countryside Mayorka. Likewise, those from Lomyvsky Forest-Park had higher contents than those of the Botanical Garden. These differences are likely driven by location-specific geomorphological and edaphic conditions. Monastyrsky Island and Lomyvsky Forest-Park are characterized by alluvial arenosols that are periodically enriched with nutrients [47, 48]. By contrast, the upland sites Botanical Garden and Mayorka consist of chernozems, which, despite their high organic matter content, can immobilize N in mineral complexes [49, 50]. In addition, Monastyrsky Island and Lomyvsky Forest-Park are closer located to industrial facilities [51] and may thus experience elevated atmospheric CO_2_ concentrations, potentially affecting photosynthesis and C accumulation in the leaflets. Local environmental conditions are well known to impact the C and N contents of leaves in various species, shaping their ecological niche [26, 52, 53]. Moreover, the N content in *R. pseudoacacia* leaflets may be affected by the rhizobia-mediated ability to fix atmospheric nitrogen. This process is also context-dependent and influenced by environmental factors, such as, for example, the availability of soil water, nitrogen and light [54, 55]. Symbiotic nitrogen fixation was not assessed in our study.

Compared to the differences between locations, the two leaf miner species had only minor effects on foliar C and N contents of *R. pseudoacacia* leaflets, with miner infestation leading to slightly enhanced contents. Likewise, in leaves of *Betula pendula*, midrib damage by *Eriocrania* spp., but not lamina damage, was associated with a trend towards increased foliar N [56]. In *Avicennia marina*, mining led to fine-tuned changes in N and C contents in mined vs. non-mined leaf portions. Here, higher N contents in non-mined compared to mined-parts of mined leaves may indicate a higher storage capacity of N in non-mined parts, but also that photosynthesis may be impaired to varying degrees in the different leaf parts [14]. Changes of C and N contents caused by mining may in turn affect the foliar nutritional quality for the leaf miners, other herbivore species and influence their predators [56, 57]. In leaflets of *R. pseudoacacia* trees with an age of 10–25 years in the Botanical Garden infested by *P. robiniella*, we observed the highest within-group variability in N content. This variability may be driven by differences in infestation levels. Positive correlations between foliar N content and herbivore damage have been reported, for example, in leaves of *Betula pubescens* spp. *tortuosa* [58].

Among the *R. pseudoacacia* trees of different age from the Botanical Garden, the metabolic fingerprints were distinct to some extent. These differences across ontogeny may reflect a combination of physiological constraints, different environmental pressures in different years or decades and/or evolutionary conserved defense strategies [59]. Young (juvenile) trees frequently exhibit more pronounced induced defenses due to higher metabolic activity, whereas mature woody plants are often more protected by physical defenses [4], partly related to increases in condensed tannins [23, 25]. Alternatively or in addition, photosynthetic activity may change at the same crown height with age, as the trees grow taller and lower leaves become more shaded by upper leaves, highlighting the pronounced intra-individual plasticity within tree species [60, 61]. In numerous forest tree species, photosynthesis declines with age [11]. Also, due to the expansion of the root system with age, resource uptake may change and concentrations of non-structural carbohydrates may increase in different plant parts [11, 62]. Such changes in defense strategies, physiology and allocation may be reflected in different leaf metabolic patterns across tree age [63, 64]. Moreover, in the current study *R. pseudoacacia* trees showed age-dependent responses to the miners, indicating that chemical defense strategies are dynamically reconfigured across the life-span of *R. pseudoacacia*. However, we cannot rule out that different infestation levels in trees of different ages contributed to our findings. Metabolic heterogeneity, as found among tree age classes in our study, may also function as a population-level defense against herbivores [65, 66].

Pronounced differences in metabolic composition were found between *R. pseudoacacia* leaflets from trees of the same age class but from different locations. Each study location exhibits distinct edaphic and microclimatic conditions, likely contributing to these divergent metabolite compositions. For example, trees exposed to variation in soil type, nitrogen deposition or moisture regimes may differentially allocate resources to specialized metabolites in the leaves [26, 67]. In our study, the metabolic fingerprints of leaflets from *R. pseudoacacia* trees on arenosol (Monastyrsky Island, Lomyvsky Forest-Park) were distinct from those of trees growing on chernozems (Mayorka, Botanical Garden). Among other aspects, these soil types differ in terms of nutrient availabilities, as discussed above. In nutrient-poor or water-limited environments, slower growth and surplus fixed C frequently promote increased investment in carbon-based specialized metabolites, such as phenolics and tannins [68, 69]. Moreover, differences in technogenic CO_2_ concentrations or pollution levels across study locations [51, 70] may have led to varying oxidative stress and nutrient balances in leaves, potentially partly explaining the observed location-specific metabolic fingerprints of leaflets. Previous studies have shown that exposure of *R. pseudoacacia* to excess CO_2_ increases levels of reactive oxygen species, disrupts the antioxidant system and affects various proteins, including those involved in the biosynthesis of specialized metabolites [71].

Compared to the differences between locations, metabolic fingerprints of *R. pseudoacacia* leaflets were modulated less by herbivory, but still some quantitative and qualitative metabolic changes were found. Some metabolic features showed higher, while others had lower concentrations under herbivory. The two invasive leaf miners elicited broadly similar changes, suggesting that shared biochemical pathways may underlie the induction patterns. However, there were also leaf miner species-specific responses of metabolic features, which may be due to slight differences in mesophyll feeding patterns between *P. robiniella* and *M. robiniella*, differences in amount of tissue damaged and/or in the feeding duration. While changes in leaf metabolites due to mining have been shown previously in different tree species [8, 56], little is known about species-specific responses to mining herbivores. Generally, in plant–miner systems, leaf mining can lead to an accumulation of phenolics and other defense compounds [8, 16, 17]. With regard to phytohormones, cytokinins seem mostly involved in plant responses to mining insects, while jasmonic and salicylic acid play subordinate roles [18, 72]. Thereby, insects may provide the plants with cytokinins, manipulate their physiology and reprogram their metabolism [72]. Overall, we cannot exclude that the air-drying of the leaves led to some changes in metabolites. Nevertheless, all samples were treated in a comparable way and various phytochemicals had also previously been identified from air-dried material of *R. pseudoacacia* by other researchers [35]. The location-specificity of foliar metabolic responses to herbivory found in our study probably reflects different capabilities to induce responses due to varying environmental conditions, including nutrient availability, but may also be related to location-specific infestation levels.

Both *P. robiniella* and *M. robiniella* are novel in the invaded range in Ukraine, but belong to *R. pseudoacacia’*s herbivore community within the native range in North America [73]. Thus, *R. pseudoacacia* may have coevolved with these insect species in its native range. This co-evolution probably explains part of the observed plant metabolic responses to the herbivores, while the conditions in the invaded range may have further shaped these responses.

## Conclusions

In summary, *R. pseudoacacia* exhibits context-dependent biochemical responses to leaf miner herbivory, with both tree age and local habitat conditions pronouncedly shaping the plant responses. Our study highlights the importance of considering both plant ontogenetic stage and local (a)biotic conditions when assessing tree vulnerability and responses to invasive herbivores. This work provides a basis for future research on specific metabolites associated with resistance to leaf miners and how environmental conditions might influence plant–leaf miner chemical interactions.

## Supplementary Information

Supplementary Material 1.

Supplementary Material 2.

## Data Availability

The C and N data, intensities of metabolic features and log2-scaled fold changes are available in Supplement Table S3. The raw LC-MS data will be made available on MetaboLights [74].
