# Supplementary material for: Biochemical changes in Robinia pseudoacacia leaflets in dependence of leaf mining, plant age and location
Source: BMC Plant Biol. 2026 Feb 14;26:387. doi: 10.1186/s12870-026-08364-6 (PMC12931043; doi:10.1186/s12870-026-08364-6)
Supplement: Supplementary file 1 — Supplementary Material 1. [file 12870_2026_8364_MOESM1_ESM.pdf]

Supplementary Material

Biochemical changes in *Robinia pseudoacacia* leaflets in dependence of leaf mining, plant age and location

Svitlana Sytnyk, Rabea Schweiger, Kyrylo Holoborodko, Caroline Müller

Table S1. Characteristics of the sampling locations of *Robinia pseudoacacia*

	**Location**			
	Botanical Garden (BotG)	Mayorka (May)	Monastyrsky Island (MonIs)	Lomyvsky Forest-Park (LomFP)
GPS latitude<br>longitude<br>Height above sea level (m)	48º43'09.57" N<br>35º04'02.64" E<br>127	48º15'49.97" N<br>35º09'47.90" E<br>104	48º27'34.02" N<br>35º04'58.01" E<br>54	48º30'36.50" N<br>34º58'54.89" E<br>54
Relief part type	watershed gully, upper third of ravine	watershed gully	Dnipro river valley, washed sandbar	Dnipro river valley
Soil type	calcic black soil, chernozem	calcic black soil, chernozem, loamy	arenosol	fluvic arenosol, sandy loam
Ground water depth (m)	30–35	20–25	0.9–1.2	2.0–2.5
Dominant tree species composition in the stand	*Acer platanoides*, *Acer negundo*, *Gleditsia triacanthos*		*Populus alba*, *Populus nigra*, *Salix alba*	*Pinus sylvestris*, *Populus alba*, *Populus nigra*
Share of *R. pseudoacacia* of all trees (%)	27	100	20	15
Functional purpose of stand	green zone in urban area	protection against soil erosion	green zone in urban area	green zone in urban area
Tree age class (years)	< 10<br>10–25<br>> 25	< 10	< 10	< 10
Tree height (m) (mean ± SD)	4.66 ± 1.15 (< 10)<br>12.10 ± 1.21 (10–25)<br>19.3 ± 1.17 (> 25)	4.37 ± 0.97	4.96 ± 0.76	5.01 ± 0.99
Stem diameter (cm)* (mean ± SD)	3.20 ± 1.02 (< 10)<br>14.88 ± 1.91 (10–25)<br>29.3 ± 3.08 (> 25)	2.99 ± 0.91	2.88 ± 0.77	3.21 ± 0.67
Categories of leaflet infestation for harvest**	uninfested<br>infested with P<br>infested with M	uninfested<br>infested with P	uninfested<br>infested with P	uninfested<br>infested with P<br>infested with M

* – tree stem diameter was measured at 1.3 m height; ** P – *Parectopa robiniella*; M – *Macrosaccus robiniella*

Table S2. Results of manual contrasts for carbon (C) and nitrogen (N) content of *Robinia pseudoacacia* leaflets.

Data subset	Response variable	Comparison (manual contrast)						P^1
		Group 1			versus Group 2			
		Location	Tree age	Herbivory	Location	Tree age	Herbivory	
I	C content	BotG	< 10 y	P	BotG	< 10 y	U	0.760
		BotG	< 10 y	M	BotG	< 10 y	U	0.760
		BotG	10-25 y	P	BotG	10-25 y	U	0.760
		BotG	10-25 y	M	BotG	10-25 y	U	0.948
		BotG	> 25 y	P	BotG	> 25 y	U	0.261
		BotG	> 25 y	M	BotG	> 25 y	U	*0.055*
		BotG	< 10 y	U	BotG	10-25 y	U	0.752
		BotG	> 25 y	U	BotG	10-25 y	U	0.760
	N content	BotG	< 10 y	P	BotG	< 10 y	U	1.000
		BotG	< 10 y	M	BotG	< 10 y	U	0.436
		BotG	10-25 y	P	BotG	10-25 y	U	**0.024**
		BotG	10-25 y	M	BotG	10-25 y	U	1.000
		BotG	> 25 y	P	BotG	> 25 y	U	1.000
		BotG	> 25 y	M	BotG	> 25 y	U	1.000
		BotG	< 10 y	U	BotG	10-25 y	U	0.435
		BotG	> 25 y	U	BotG	10-25 y	U	1.000
IIa	C content	May	< 10 y	P	May	< 10 y	U	0.254
		MonIs	< 10 y	P	MonIs	< 10 y	U	0.254
		May	< 10 y	U	MonIs	< 10 y	U	**<0.001**
	N content	May	< 10 y	P	May	< 10 y	U	0.941
		MonIs	< 10 y	P	MonIs	< 10 y	U	0.941
		May	< 10 y	U	MonIs	< 10 y	U	*0.053*
IIb	C content	BotG	< 10 y	P	BotG	< 10 y	U	0.436
		BotG	< 10 y	M	BotG	< 10 y	U	0.806
		LomFP	< 10 y	P	LomFP	< 10 y	U	0.806
		LomFP	< 10 y	M	LomFP	< 10 y	U	0.806
		BotG	< 10 y	U	LomFP	< 10 y	U	**0.001**
	N content	BotG	< 10 y	P	BotG	< 10 y	U	0.351
		BotG	< 10 y	M	BotG	< 10 y	U	**0.035**
		LomFP	< 10 y	P	LomFP	< 10 y	U	1.000
		LomFP	< 10 y	M	LomFP	< 10 y	U	1.000
		BotG	< 10 y	U	LomFP	< 10 y	U	*0.099*

[1] significant *p*-values (< 0.05) are indicated in bold, marginally significant ones (< 0.1) in italics

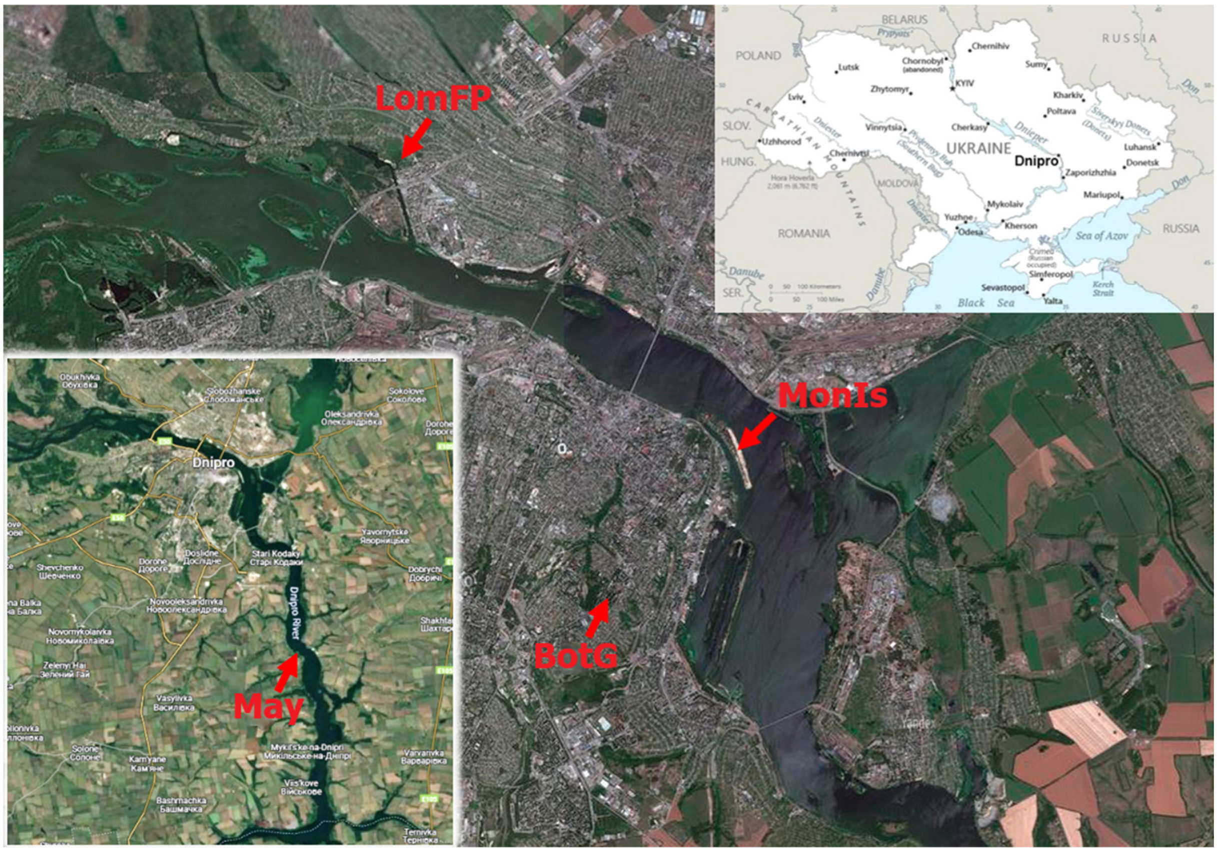

Fig. S1 Sampling region around Dnipro, south-eastern Ukraine. BotG, Botanical Garden; May, Mayorka; MonIs, Monastyrsky Island; LomFP, Lomyvsky Forest-Park. Map in upper right corner taken from The National Atlas of Ukraine (2007), the other two maps are taken from Google Maps.

Reference: National Atlas of Ukraine (2007) L. H. Rudenko (Ed. in chief), B. Ye. Paton (Head of editorial board). Kyiv: DNVP Kartographia.

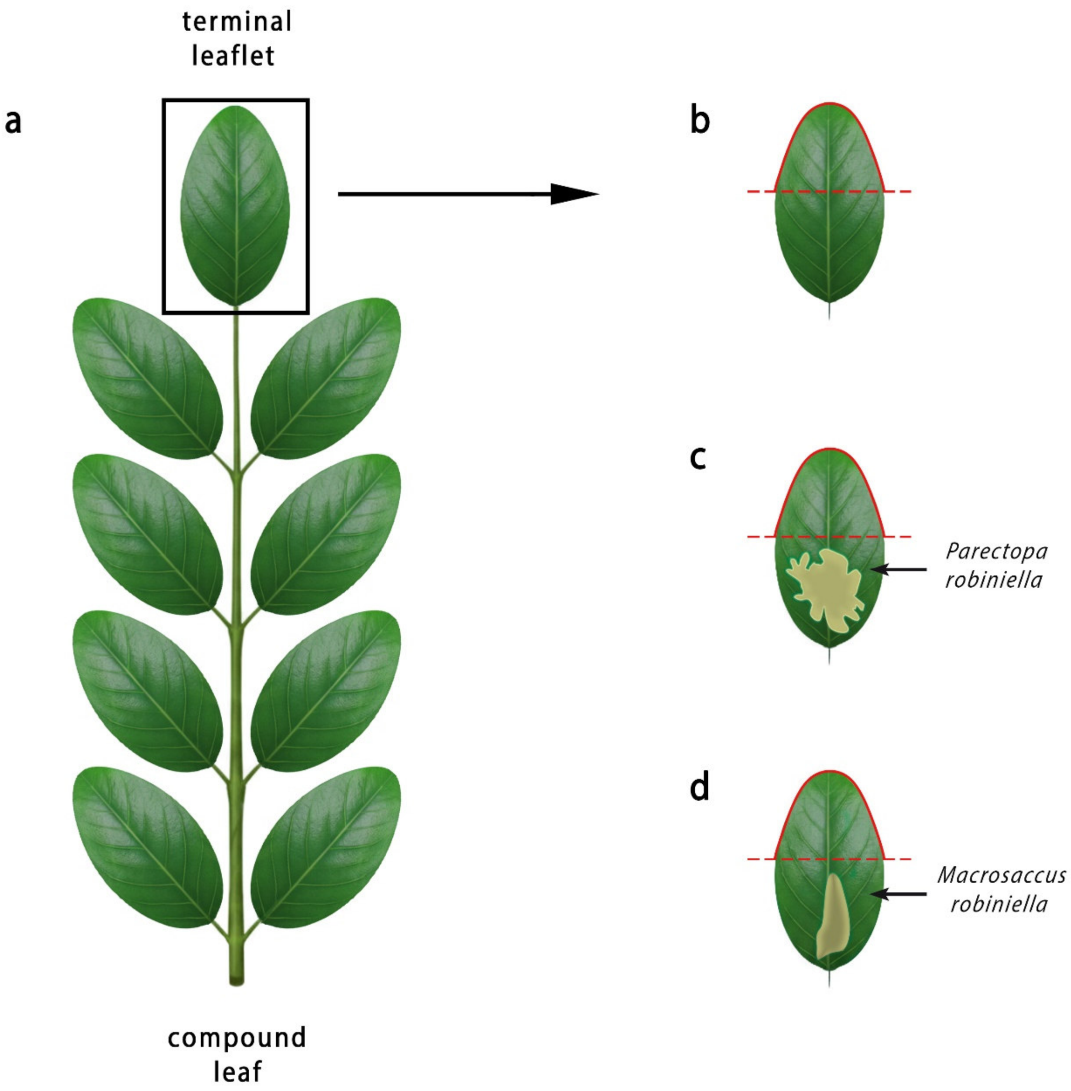

Fig. S2 Compound leaf of *Robinia pseudoacacia* with the terminal leaflets indicated with a box (**a**). Terminal leaflets were either uninfested (**b**) or mined by larvae of *Parectopa robiniella* (**c**) or *Macrosaccus robiniella* (**d**). The harvested sections are indicated in red.

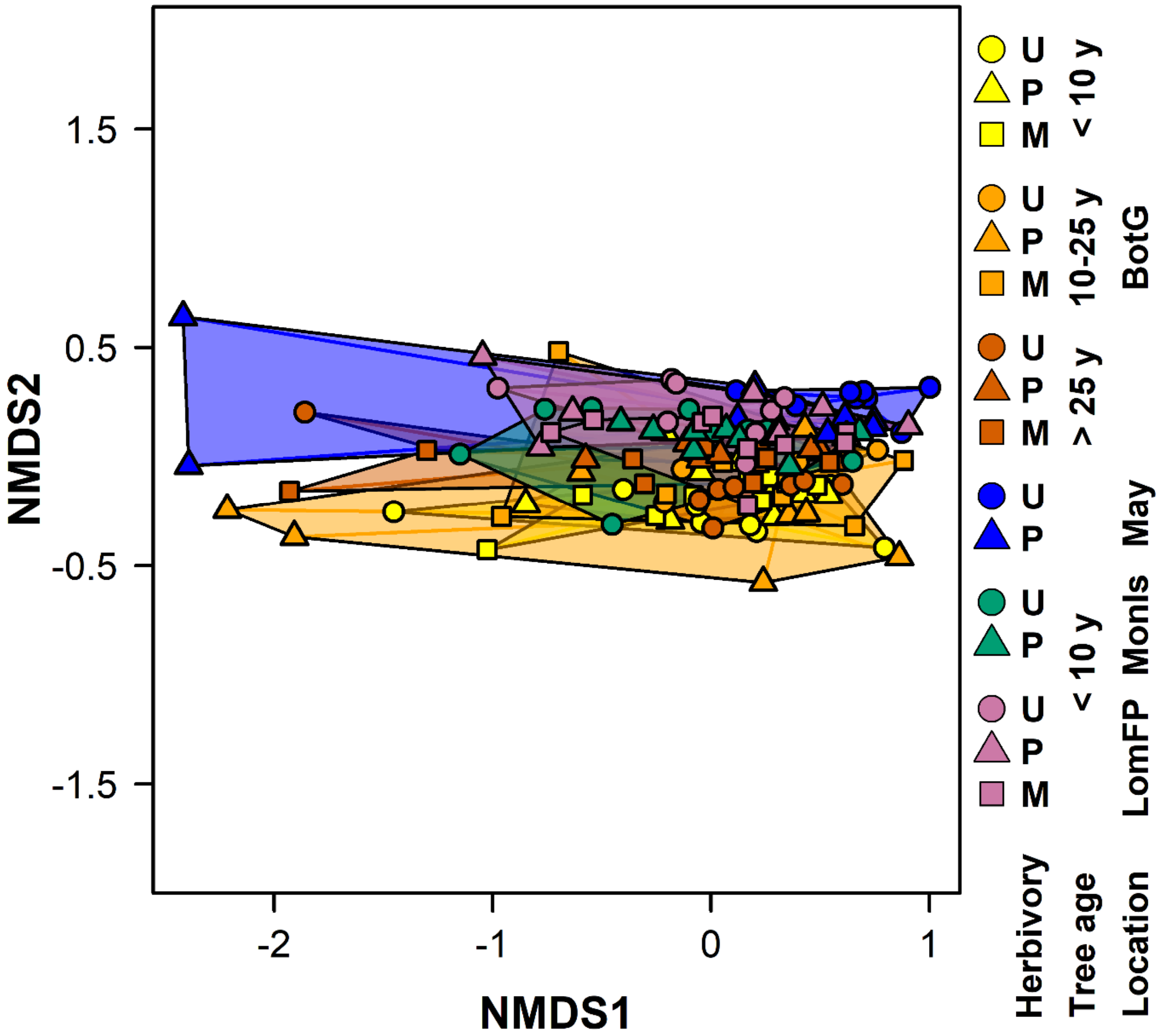

Fig. S3 Non-metric multidimensional scaling (NMDS; stress value 0.102) plot showing the metabolic composition of *Robinia pseudoacacia* leaflets. Leaflets were uninfested (U) or infested by herbivores [*Parectopa robiniella* (P), *Macrosaccus robiniella* (M)]. Data are shown for different tree age classes and locations (BotG, Botanical Garden; May, Mayorka; MonIs, Monastyrsky Island; LomFP, Lomyvsky Forest-Park). The analysis is based on 3,121 metabolic features. The groups are surrounded by convex hulls and scores are connected to the group medians; n = 7-10.

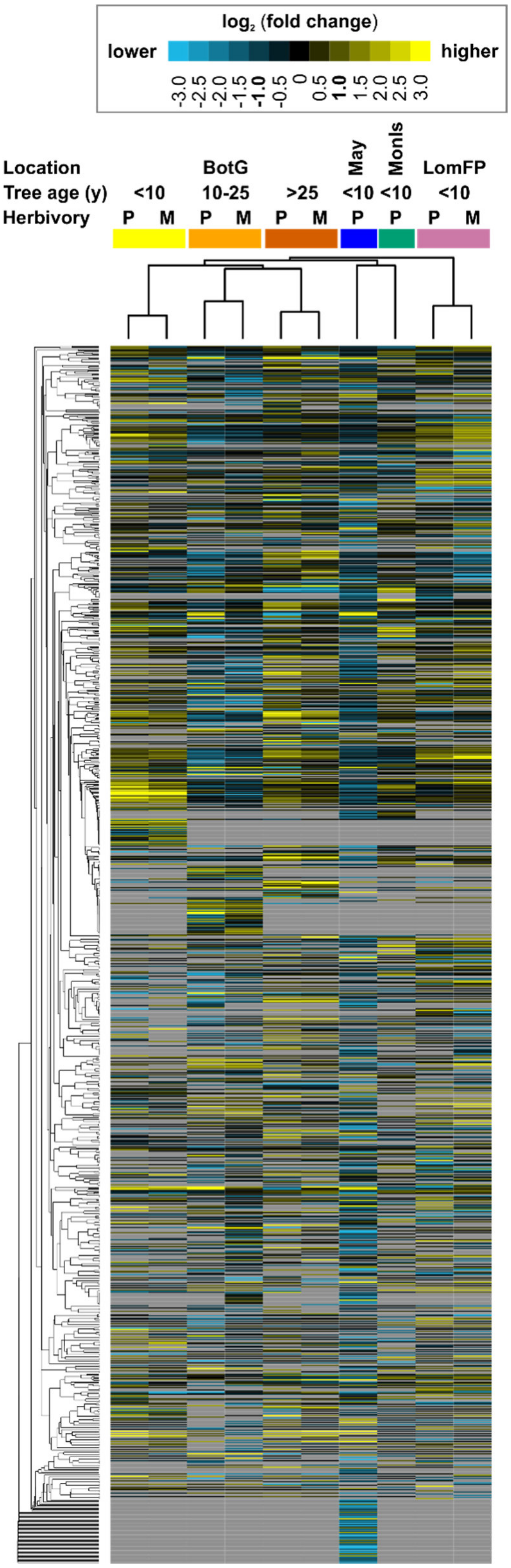

Fig. S4 Cluster heatmap of metabolic responses of *Robinia pseudoacacia* leaflets to herbivory. For different tree age classes and locations (BotG, Botanical Garden; May, Mayorka; MonIs, Monastyrsky Island; LomFP, Lomyvsky Forest-Park), responses to infestation by *Parectopa robiniella* (P) or *Macrosaccus robiniella* (M) are shown as mean fold changes (log_2-scaled) of metabolic features in herbivore-infested leaflets compared to uninfested leaflets. The heatmap

is based on those 1,087 metabolic features, which occurred in both groups and were quantitatively modulated ($\log_2$ fold change < -1 or > 1) in at least one pairwise comparison (for raw data, see Table S3). Yellow indicates higher concentrations of features under herbivory, while blue indicates lower concentrations under herbivory. In the color legend, the fold change thresholds that were used to define features as being quantitatively modulated by herbivory are given in bold. Gray means that the corresponding feature was not detected in one or both of the groups compared; means based on n = 7-10.
